# Metaphylogenomic and Potential Functionality of the Limpet *Patella pellucida*’s Gastrointestinal Tract Microbiome

**DOI:** 10.3390/ijms151018819

**Published:** 2014-10-20

**Authors:** Magda Dudek, Jessica Adams, Martin Swain, Matthew Hegarty, Sharon Huws, Joe Gallagher

**Affiliations:** Institute of Biological, Environmental & Rural Sciences (IBERS), Aberystwyth University, Gogerddan, Aberystwyth, Ceredigion, Wales SY23 3EE, UK; E-Mails: mad27@aber.ac.uk (M.D.); jaa@aber.ac.uk (J.A.); mts11@aber.ac.uk (M.S.); ayh@aber.ac.uk (M.H.); hnh@aber.ac.uk (S.H.)

**Keywords:** *Patella pellucida*, limpet, mollusc, microbes, symbiosis, metagenomics, bioenergy, biorefining, seaweed, macroalgae

## Abstract

This study investigated the microbial diversity associated with the digestive tract of the seaweed grazing marine limpet *Patella pellucida*. Using a modified indirect DNA extraction protocol and performing metagenomic profiling based on specific prokaryotic marker genes, the abundance of bacterial groups was identified from the analyzed metagenome. The members of three significantly abundant phyla of *Proteobacteria*, *Firmicutes* and *Bacteroidetes* were characterized through the literature and their predicted functions towards the host, as well as potential applications in the industrial environment assessed.

## 1. Introduction

**Figure 1:**
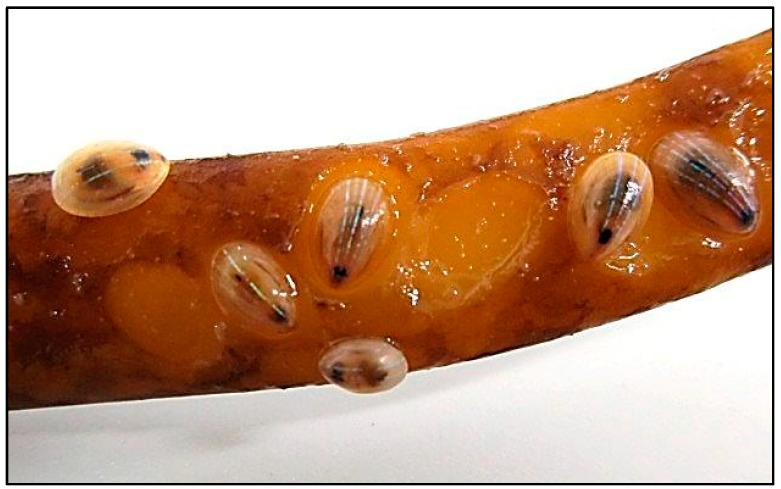
*P. pellucida* grazing on a stem of *L. digitata*.

*Patella pellucida* (Linnaeus, 1758), commonly known as the blue-rayed limpet or peacock’s feathers [1], is a key seaweed grazer growing up to 15 mm in length and present on almost all Atlantic European coasts [2]. This small mollusc is a parasite of brown algae (mainly *Laminaria digitata*) and is often found buried in a self-digested hole within the stem of the seaweed (Figure 1). Brown algae are the main diet of the blue-rayed limpet. This limpet begins its life cycle when the seaweed accumulates high levels of sugars and ends when level of sugars decrease [3]. In order to digest and assimilate the polymeric carbohydrates present in the seaweed, including alginic acid, laminarin, fucoidan and cellulose [4], the limpet has developed very efficient enzymatic systems that, as with most herbivorous land and marine animals [5,6,7], is likely to involve a contribution by symbiotic microorganisms present in the digestive tract. Living in the open sea waters and being continuously exposed to predation, adult *P. pellucida* and its larvae are also likely to be protected by a chemical defense mechanism, often originated from stable endogenous bacterial communities [8].

Symbiotic microorganisms associated with marine animals, both invertebrates such as sea snails, sea cucumbers, sea urchins [9,10,11] as well as vertebrates e.g., marine iguanas or sea cows [6,7], have recently become a focus for research leading to the discovery of various bioactive compounds exploitable by industry. By possessing a large arsenal of enzymes which display unique properties, e.g., stability in high salt concentrations, adaptation to cold temperatures, extreme pH tolerance as well as specificity for a broad range of substrates [12], some of these beneficial microbes have the potential to improve the efficiency of biomass conversion, a main bottleneck in today’s biorefinery processes [13]. Particularly with the rapid development of blue biotechnology, based on marine-derived feedstock (e.g., micro and macro algae, waste from seafood processing), there is a growing interest in the applications of such microbial biocatalysts [14]. Symbiotic microorganisms, originally defending their marine animal host from predators, are now also seen as a potential source of novel drugs including new forms of antibiotics and anticancer treatments [15]. With new discoveries coming to light every year, microbial symbiosis in the marine ecosystem appears to be an untapped source of many other bio-compounds, which can be now better studied thanks to advanced molecular methods such as metagenomics [16].

Recent developments in using metagenomics offer a powerful alternative to culture dependent methods, providing an opportunity to study genomes of microbes that to date are uncultivable in the laboratory [17]. High-throughput metagenomic approaches have given great insights into the diversity, and function of microbial communities hosted by marine animals, expanding our knowledge on the biotechnological potential of prokaryotic populations inhabiting these hardly accessible, host-dependent niches [11,18,19]. Many metagenomic projects are based on an initial and crucial step which involves extraction of DNA [20]. Obtaining good quality microbial metagenomic DNA from samples where the target community is associated with an animal host (such as *P. pellucida*) can be problematic. This is mainly due to the contamination of prokaryotic material with eukaryotic cell-derived nucleic acids, introducing a significant bias in the analysis of the metagenomic reads, leading to the underestimation of microbial community size and composition [21]. The successful construction of metagenomic libraries from environmental samples therefore often relies on targeted cell separation prior to DNA extraction, which should not only reduce contamination by host DNA but also provide a high molecular weight and a satisfactory recovery rate of output DNA. This process, called indirect DNA extraction has several clear advantages over direct extraction of total DNA, such as yielding longer fragments of DNA, improving its purity and avoiding “noise” in the metagenomic sequence reads [22]. Reports in the literature describe various attempts to separate microbial communities from environmental samples using, e.g., density gradients [18], gel electrophoresis [23] or filtration [24]. None of these methods have been suitable to date as a universal protocol for indirect DNA extraction and there is always a risk that their application may result in underestimation of microbial diversity within the sample.

In this study a modified protocol for the indirect extraction of prokaryotic DNA was developed and applied to samples derived from *P. pellucida*’s digestive tract. Extracted DNA was used for the construction of shotgun metagenomic libraries, which have been sequenced and analysed using Metagenomic Phylogenetic Analysis (MetaPhlAn), a powerful, new taxonomic classifier based on prokaryotic clade-specific marker genes [25,26]. Metagenomic analysis of the phylogenetic profiles and a prediction of the microbial functional roles in *P. pellucida* is the first step in assessing their potential for exploitation in the industrial environment.

## 2. Results and Discussion

**Figure 2:**
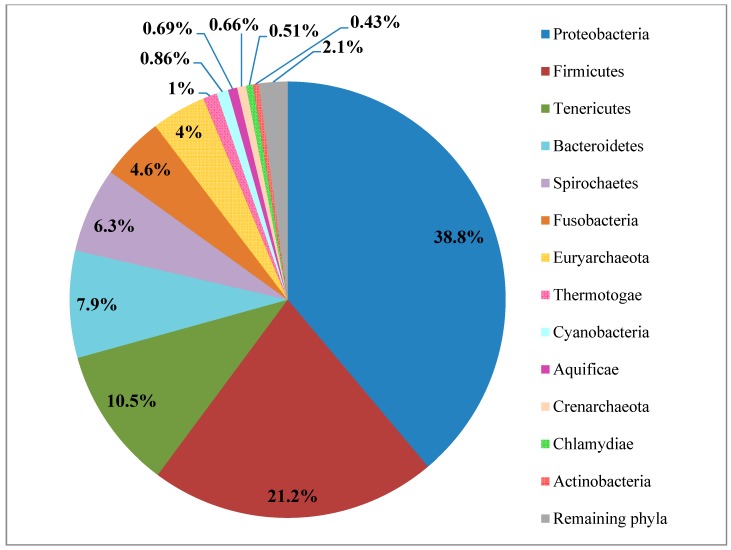
Phylum level classification of the *P. pellucida* gastrointestinal tract microbiome where the percentages are the relative abundances estimated by MetaPhlAn.

Results of the MetaPhlAn analysis revealed a diverse microbial community in the *P. pellucida*’s gastrointestinal tract (Figure 2). The predominant phylum in the microbial metagenome was the phylum *Proteobacteria*, with 38.8% relative abundance. The second predominant bacterial lineage, constituting 21.2%, was identified as phylum *Firmicutes* and was followed by *Tenericutes*, *Bacteroidetes* and *Spirochaetes*, accounting respectively for 10.5%, 7.9% and 6.3% relative abundance. Less prevalent phyla were the *Fusobacteria* (4.6%) and archaeal phylum *Euryarcheota* (4%). Finally, representing <1% of the total abundance, were phyla such as *Thermotogae*, *Cyanobacteria*, *Aquificae*, *Crenarcheota*, *Chlamydiae*, *Actinobacteria* and others. 

**Figure 3:**
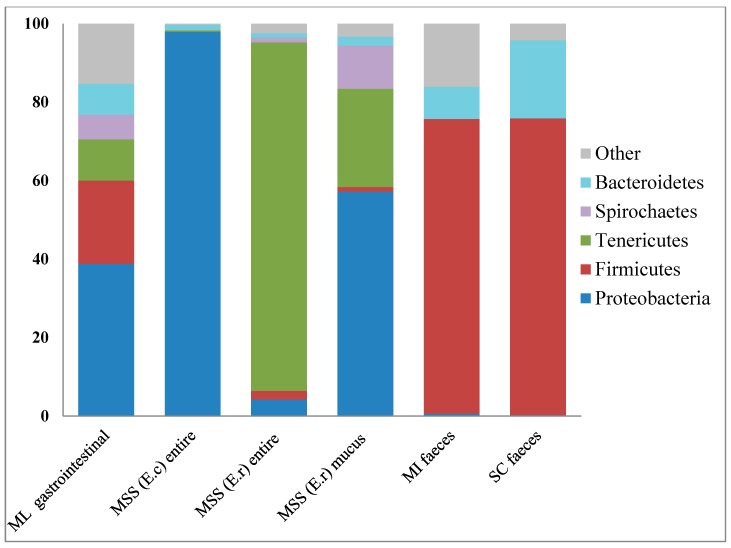
Comparison of the Phylum level microbiome associated with *P. pellucida* gastrointestinal tract to those associated with other seaweed and sea grass grazers: ML-Marine Limpet *P. pellucida*; MSS (E.c)-Marine Sea Slug *Elysia chlorotica*; MSS (E.r)-Marine Sea Slug *Elysia rufescens*; MI-Marine Iguana *Amblyrhynchus cristatus*; SC-Sea Cow *Dugong dugon*.

**Figure 4:**
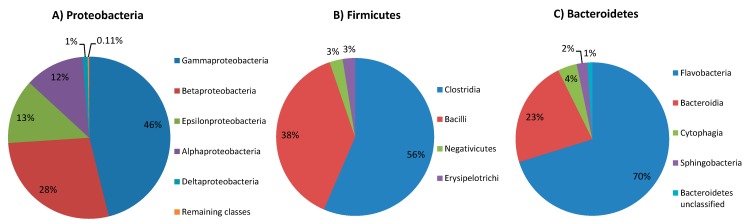
Phylogenomic class level characterization within the phyla (**A**) *Proteobacteria* (**B**) *Firmicutes* and (**C**) *Bacteroidetes* (from the digestive tract of *P. pellucida*).

To our current knowledge only a few microbiomes associated with seaweed or sea grass eating animals have been analyzed in metagenomic projects (Figure 3). Previous metagenomic studies investigated the microbial assemblage from the sea slug *Elysia chlorotica* [27], its relative *Elysia rufescens* [28], the marine iguana *Amblyrhynchus cristatus* [6] and the sea cow *Dugong dugon* [7]. Of these, only the two last metagenomes could be directly related to the diet of their animal hosts as metagenomic data was generated based on microbial DNA extracted from faeces of marine iguana and sea cow, respectively. In the case of both molluscs, metagenomic analysis of microbial communities revealed main groups of microbes associated with the entire body of the slugs as well as the mucus from *Elysia rufescens*. Based on this data and literature concerning the industrial potential of marine microbes we characterized the microbiome from *P. pellucida*’s gastrointestinal tract focusing on three bacterial phyla: *Proteobacteria*, *Firmicutes* and *Bacteroidetes* (Figure 4A–C). These three phyla were investigated due to their significant abundance within the sample as well as the biotechnological potential that certain of their members could present and which therefore deserves to be explored in further research.

### 2.1. Proteobacteria

The taxonomic analysis of metagenomic reads indicated *Proteobacteria* as the most abundant phylum in the digestive tract of *P. pellucida* (Figure 2). This phylum is currently the largest and the most complex phenotypically bacterial lineage, which usually dominates prokaryotic taxa in samples derived from marine environments and is very often associated with marine animals including sea snails (Figure 3), fish, shrimps and sponges [19,27,28,29,30]. Due to the huge biodiversity, members of this phylum have been shown to display various functions towards the specific marine ecosystem where they are found. Many of those features such as production of powerful enzymes, synthesis of antimicrobial compounds or antifouling agents have been identified [8,31], but much more is yet to be discovered.

MetaPhlAn analysis indicated that the majority of *Proteobacteria* harbored by the limpet was composed of the class *Gammaproteobacteria* as well as four less abundant subdivisions: *Betaproteobacteria*, *Epsilonoproteobacteria*, *Alphaproteobacteria* and *Deltaproteobacteria* (Figure 4A). The majority of shotgun reads within phylum *Proteobacteria* were assigned to the group of uncultivated endosymbionts with reduced genomes (Table 1) such as those belonging to the genus “*Candidatus* Carsonella” [32] (class *Gammaproteobacteria*) and “*Candidatus* Zinderia” [33] (class *Betaproteobacteria*). Being vertically transferred with eggs from one host generation to another these bacteria are known to live inside bacteriocytes providing their hosts with essential compounds in exchange for nutrients and protection [34]. The large number of metagenomic reads identified as belonging to these microbes could be explained by the fact that metagenomic DNA was isolated from *P. pellucida* when the limpets were in their reproductive cycle. Due to the small size and proximity of organs composing the visceral mass of *P. pellucida*, dissected gastrointestinal material was most probably contaminated with symbiotic bacteria residing in developing eggs. Related microbes to these, e.g., “*Candidatus* Endobugula sertula”, found in larvae of marine bryozoan *Bugula neritina* [35], have recently been identified as a likely source of bryostatins. These are natural polyketide compounds providing defense against predators and have been extensively tested for anticancer activity [36]. In its life cycle *P. pellucida* also undergoes a planktonic larvae stage [3], possibly with a similar chemical defense protection mechanism. Thus it is possible that genes for synthesizing novel polyketide toxins could be found in abundant populations of related intracellular symbionts harbored by the limpet.

Another group of *Proteobacteria*, distinguished based on MetaPhlAn analysis, was represented by bacteria that are known to display chemolithotrophic properties (Table 1). Analysis predictions identified sequences for microbes in the *Betaproteobacteria* class assigned to the genus *Nitrosomonas*. This genus is known to include ammonia-oxidizing bacteria. Another class, *Epsilonoproteobacteria* included bacteria belonging to sulphur and nitrate reducing genera such as *Nitratiruptor* and *Caminibacter*, whereas class *Deltaproteobacteria* was dominated by sulphate-reducing microbes from the genera *Desulfovibrio* and *Desulfotalea*. Interestingly, certain members of these genera are known from the literature to be strictly thermophilic microbes associated with extreme aquatic environments, e.g., hydrothermal vents as well as the invertebrates thriving there [37,38,39], and their presence in association with the cold water mollusc *Patella pellucida* appears enigmatic. Some of these bacteria may be displaying an ability for efficient neutralization of toxic metal ions (e.g., *Desulfovibrio vulgaris*) [40] or the degradation of a variety of halogenated organic compounds (e.g., *Nitrosomonas europaea*) [41]. These microbes and their abilities could be of special interest for industrial applications such as bioremediation of oil-polluted marine environments or contaminated soils.

**Table 1:** Categorization and predicted function of members of the phylum *Proteobacteria*.

Class	The Most Abundant Order (% of Class)	The Most Abundant Family (% of Order)	The Most Abundant Genus/Species	Functional Role/Habitat	Ref.
*Gammaproteobacteria*	*Enterobacteriales* (76.34)	*Enterobacteriaceae* (100)	“ *Candidatus* Carsonella”/ “*Candidatus* Carsonella rudii”	nutrients supply/obligate endosymbiont of psyllids	[32,34]
	*Pasteurellales* (4.84)	*Pasteurellaceae* (100)	*Haemophilus*/ *Haemophilus influenzae*	pathogenic/human and animals	[42]
	*Thiotrichales* (3.45)	*Francisellaceae* (87.09)	*Francisella*/ *Francisella tularenisis*		[43]
*Betaproteobacteria*	*Burkholderiales* (94.65)	*Oxalobacteraceae* (98.63)	“ *Candidatus* Zinderia”/ “*Candidatus* Zinderia” (unclassified)	nutrients supply/obligate endosymbiont of spittlebug	[33]
	*Neisseriales* (4.7)	*Neisseriaceae* (100)	*Neisseria*/ *Neisseria meningitides*	pathogenic/human origin	[44]
	*Nitrosomonadales* (0.27)	*Nitrosomonadaceae* (100)	*Nitrosomonas*/ *Nitrosomonas europea*	ammonia oxidation/sewage plants disposal; water; soil	[41,45]
*Epsilonoproteobacteria*	*Campylobacterales* (81.6)	*Campylobacteraceae* (64.21)	*Campylobacter*/ *Campylobacter lari*	pathogenic/gastrointestinal of human and animals	[46]
	*Nautiliales* (16.6)	*Nautilaceae* (100)	*Caminibacter*/ *Caminibacter mediatlanticus*	nitrate and sulphur reduction/deep-sea hydrothermal systems	[37]
	*Epsilonoproteo-bacteria* (unclassified) (1.72)	*Nitratiruptor* (62.5)	*Nitratiruptor*/ *Nitratiruptor* (unclassified)		[38]
*Alphaproteobacteria*	*Rickettsiales* (76.67)	*Rickettsiaceae* (60.5)	*Rickettsia*/ *Rickettsia bellii*	pathogenic/ human and animals	[47]
	*Rhizobiales* (19.65)	*Bartonellaceae* (45.05)	*Bartonella*/ *Bartonella henselae*	pathogenic/ human and animals	[48]
		*Brucellaceae* (38.46)	*Brucella*/ *Brucella abortus*		[49]
*Deltaproteobacteria*	*Desulfovibrionales* (41.02)	*Desulfovibrionaceae* (87.5)	*Desulfovibrio*/ *Desulfovibrio magneticus*	sulphate-reduction/marine sediments; gastrointestinal of human and animals	[50,51]
	*Desulfobacterales* (17.94)	*Desulfobulbaceae* (42.85)	*Desulfotalea*/*Desulfotalea psychrophila*		[52]
	*Bdellovibrionales* (17.94)	*Bacteriovoraceae* (85.7)	*Bacteriovorax*/ *Bacteriovorax marinus*	predatory/marine environment	[53]

*Proteobacteria* associated with the gastrointestinal tract of *P. pellucida* were also comprised of many species belonging to genera associated with pathogens of marine animals and plants and known to cause foodborne human disease. Of these, bacteria from genera *Haemophilus* and *Francisella* were identified in the *Gammaproteboacteria* class; members of *Neisseria* were found in the *Betaproteobacteria* class; species belonging to the genus *Campylobacter* in the *Epsilonoproteobacteria* class and species from genera: *Rickettsia*, *Bartonella* as well as *Brucella* in the *Alphaproteobacteria* class.

### 2.2. Firmicutes

Bacteria from the phylum *Firmicutes* were identified by MetaPhlAn metagenomic analysis as the second largest lineage in the investigated microbiome after *Proteobacteria* (Figure 2). *Firmicutes* are a group of mostly gram positive, spore forming bacteria, often anaerobic and as such are associated with environments of oxygen deficit. Microbes belonging to this phylum commonly inhabit marine ecosystems, where they can be found free living in sea water, marine sediments and as symbionts or parasites hosted by marine animals [54]. *Firmicutes* are a natural gut microbiota component of saltwater animals including marine iguanas with an exclusively algal diet [6] and sea grass-grazing sea cows [7]. In metagenomic studies on these animals they were found as the predominant phylum in faecal samples (Figure 3). Being involved in complex enzymatic processes of recalcitrant polysaccharide degradation and fermentation as well as displaying capabilities to survive a range of environmental conditions; some members of *Firmicutes* are currently among the most broadly used candidates for various biorefining processes [55,56,57,58]. Our metagenomic analysis revealed that *Firmicutes* derived from the gastrointestinal system of limpets were overrepresented by two main classes: *Clostridia* and *Bacilli*, whereas the rest of the phylum constituted two minor subdivisions: *Negavicutes* and *Erysipelotrichi* (Figure 4B). *Clostridiales* and *Thermoanaerobacterales* identified in the *Clostridia* class, as well as *Bacillales* and *Lactobacilliales* assigned to the *Bacilli* class (Table 2), represented genera and species that are broadly associated with applications in all the key metabolic stages of the biorefinery processes: polymer hydrolysis, sugar fermentation and anaerobic digestion. For example specific species of the genus *Bacillus*, found in the analysed metagenome could have similar properties to recently reported strains of *Bacillus* used for initial biological saccharification and fermentation of seaweed [59,60]. Although not usually associated with high salt concentration environments, marine bacteria from the genus *Lactobacillus* are also of growing biotechnological interest in the functional food sector due to their potential capacity to produce lactic acid from seaweed [61,62].

**Table 2:** Categorization and predicted function of members of the phylum *Firmicutes*.

Class	The Most Abundant Order (% of Class)	The Most Abundant Family (% of Order)	The Most Abundant Genus/Species	Functional Role/Habitat	Ref.
*Clostridia*	*Clostridiales* (72.62)	*Clostridiaceae* (49.71)	*Clostridium*/ *Clostridium butyricum*	polysaccharides degradation and fermentation; pathogenic/gastrointestinal of human and animals; feaces; soil; water	[54,63]
		*Clostridiales Family XI Incertae sedis* (47.23)	*Anaerococcus*/ *Anaerococcus vaginalis*	polysaccharides degradation and fermentation/clinical specimens of human origin	[64]
	*Thermoanaerobacterales* (22.71)	*Thermoanaero- bacterales Familly III Incertae sedis* (53.84)	*Caldicellulosiruptor/Caldicellulosiruptor kronotskyensis*	polysaccharides degradation and fermentation/hot springs; deep-sea hydrothermal systems	[65]
		*Thermoanaero- bacteraceae* (45.42)	*Thermoanaerobacter*/*Thermoanaerobacter ethanolicus*		[66]
*Bacilli*	*Lactobacillales* (51.96)	Streptococcaceae (40.89)	*Streptococcus*/ *Streptococcus bovis*	pathogenic/clinical specimens of human and animals origin	[67,68]
		*Lactobacillaceae* (34.51)	*Lactobacillus*/ *Lactobacillus iners*	polysaccharides fermentation/gastrointestinal of human and animals; water; soil	[61,62]
	*Bacillales* (48.03)	*Bacillaceae* (36.82)	*Bacillus*/*Bacillus thuringiensis*	polysaccharides degradation and fermentation/ gastrointestinal of human and animals; water; soil	[69]
		*Staphylococcaceae* (34.78)	*Staphylococcus*/ *Staphylococcus hominis*	polysaccharides fermentation; pathogenic/water; soil; clinical specimens of human and animals origin	[70,71]
*Negativivicutes*	*Selenomonadales* (100)	*Veillonellaceae* (96.49)	*Dialister*/*Dialister microaerophilus*	fermentation/water; soil; gastrointestinal of human and animals; clinical specimens of human origin	[72]
*Erisipelotrichi*	*Erisipelotrichales* (100)	*Erisipelotrichaceae* (100)	*Coprobacillus*/*Coprobacillus* sp. (unclassified)	fermentation/gastrointestinal of human and animals origin	[73]

### 2.3. Bacteroidetes

The third relatively abundant and biotechnologically important phylum within the prokaryotic assemblage of the marine limpet digestive tract was the *Bacteroidetes* (Figure 2). In a similar manner to the *Firmicutes*, members of this group are commonly found in marine biotopes and the intestines of marine animals. Based on the findings of previous studies, *Bacteroidetes* are often associated with microbial populations residing on seaweed [74] as well as in the guts of animals grazing on seaweed [6] or sea grass [7] (Figure 3), where one of their main functions is the degradation of high molecular weight compounds [51]. *Bacteroidetes* in marine environments are well equipped with specific mechanisms (such as adhesion proteins and genes for gliding motility) [75], allowing them to attach to the surface of seaweed, plankton or various biofilms. These features help them to get better access to the decomposed organic matter that they generate through the secretion of a range of extracellular enzymes. Due to the high plasticity of the *Bacteroidetes* genomes, involving various genetic rearrangements, gene duplications and lateral gene transfer, these bacteria can easily adapt to distinct ecological niches [76]. These facts can suggest that free-living *Bacteroidetes* consumed by *P. pellucida* could continuously contribute to the degradation of brown algae polysaccharides in the gastrointestinal tract of the limpets.

According to MetaPhlAn the majority of *Bacteroidetes* reads were assigned to the class *Flavobacteriia* followed by the class *Bacteroidia*, *Cytophagia*, *Sphingobacteriia* and unclassified *Bacteroidetes* (Figure 4C). Most of the bacteria distributed among these subdivisions were identified as belonging to the genera including bacteria of strictly saccharolytic profiles, e.g., *Cellulophaga*, *Bacteroides* or *Cytophaga* (Table 3). The recent sequencing of genomes of their members confirms that they are encoding a plethora of enzymes active towards very specific substrates. For example, examination of the *Cellulophaga lytica* type strain (LIM-21) genome revealed the existence of genes involved in degradation of cellulose, alginate and sulphated fucans [77]. The *Bacteroides thetaiotaomicron* genome was reported to encode genes catalyzing cellulose, starch, xylose, laminarin, alginate, and chitin breakdown [78], whereas the genome of *Cytophaga hutchinsonii* was predicted to encode cellulose, xylan and alginate depolymerizing enzymes [79]. *Bacteroidetes* also contained a large proportion of bacteria belonging to the genera known as obligate endosymbionts, e.g., “*Candidatus* Sulcia”, *Blattabacterium* and “*Candidatus* Amoebophilus” (Table 3), which similarly to those found in the phylum *Proteobacteria* are characteristic for their extremely reduced genomes and are predicted to provide essential nutrients to the host.

**Table 3:** Categorization and predicted function of members of the phylum *Bacteroidetes*.

Class	The Most Abundant Order (% of Class)	The Most Abundant Family (% of Order)	The Most Abundant Genera/Species	Functional Role/Habitat	Ref.
*Flavobacteriia*	*Flavobacteriales* (100)	*Flavobacteriales* (unclassified) (54)	“ *Candidatus* Sulcia”/ “*Candidatus* Sulcia muelleri”	nutrients supply/obligate endosymbiont of sharpshooters	[33]
		*Flavobacteriaceae* (36)	*Cellulophag*a/ *Cellulophaga lytica*	polysaccharides degradation/diatoms; algae; seawater	[77]
		*Blattabacteriaceae* (10)	*Blattabacterium*/ *Blattabacterium* sp. (unclassified)	nutrients supply/obligate endosymbiont of cockroaches and termites	[80]
*Bacteroidia*	*Bacteroidales* (100)	*Bacteroidaceae* (52)	*Bacteroides*/ *Bacteroides Xylanisolvens*	polysaccharides degradation and fermentation/human and animals gastrointestinal	[78,81]
		*Prevotellaceae* (24)	*Prevotella*/ *Prevotella amnii*	polysaccharides degradation and fermentation/human and animals gastrointestinal	[51,82]
		*Porphyromonadaceae* (14)	*Paludibacter*/ *Paludibacter propionicigenes*	polysaccharides fermentation/plant residue	[83]
*Cytophagia*	*Cytophagales* (100)	*Cytophagaceae* (54)	*Cytophaga*/*Cytophaga hutchinsonii*	polysaccharides degradation/soil	[79]
		*Flammeovirgaceae* (26)	*Marivirga*/ *Marivirga tractuosa*	polysaccharides degradation/water; mud; sand	[84]
		*Cyclobacteriaceae* (24)	*Algoriphagus*/ *Algoriphagus unclassified*	polysaccharides degradation/marine solar saltern	[85]
*Sphingobacteriia*	*Sphingobacteriales* (100)	*Sphingobacteriaceae* (92)	*Pedobacter*/ *Pedobacter saltans*	sulphates degradation/soil; water; fish	[86]
			*Sphingobacterium*/ *Sphingobacterium spiritivorum*	synthesis of antimicrobials/specimens of human origin	[87]
			*Mucilaginibacter*/ *Mucilaginibacter paludis*	polysaccharides degradation/sphagnum peat bog	[88]
		*Chitinophagacea* (7)	*Chitinophaga*/ *Chitinophaga pinensis*	polysaccharides degradation/soil	[89]
*Bacteroidetes* unclasified	*Bacteroidetes* (unclassified) (100)	*Bacteroidetes* (unclassified) (100)	“ *Candidatus* Amoebophilus”/ “*Candidatus* Amoebophilus” (unclassified)	nutrients supply/obligate endosymbiont of amoeba	[76]

### 2.4. Remaining Phyla

The remaining phyla within the analyzed metagenome were represented by two archaeal and eight bacterial taxons (Figure 2). Archaea identified in the gastrointestinal tract of limpets belonged to two phylogenetic lineages: *Euryarcheota* and *Crenarcheota*, encompassing respectively 4% and 0.66% of the whole microbiome. Members of both phyla were reported to be found in salt water ecosystems and are common inhabitants of certain marine animals such as sea cucumbers, sponges and fish [90,91,92]. They include extremophilic halophiles and thermophiles which produce methane (*Euryarcheota*) and are capable of ammonia oxidation (*Crenarcheota*). Of the remaining metagenomic bacteria, *P. pellucida* harbored a relatively abundant population of *Tenericutes* (10.5%), and *Spirochaetes* (6.3%), which were both previously found to be associated with the metagenome of marine sea slugs [27,28] (Figure 3). Microbes representing these phyla are primarily known as pathogens in a wide range of mammalian hosts [93,94]. Although there is a general lack of information on the function of these bacteria towards inhabited marine and freshwater animals, recent studies suggest that their members are providing benefits rather than causing detriment to their hosts which include snails, oysters and crabs [95,96]. The *Fusobacteria* phylum was found to compose 4.6% of *P. pellucida*’s microbiome and is another lineage of bacteria. This phylum has primarily been studied in relation to human and higher animals’ diseases [97], and their role in association with animals of aquatic origin is poorly understood [98]. Phyla which did not exceed 1% of total abundance were identified as: *Thermotogae*, *Cyanobacteria*, *Aquificae*, *Chlamydiae*, and *Actinobacteria,* among others. In spite of the low abundance in the metagenome, members of these microbial groups could play important roles within the blue-rayed limpet. For example, bacteria from the phylum *Thermotogae* together with the closely branched phylum of *Aquificae* could benefit the host by degrading dietary complex carbohydrates [99], members of the phylum *Actinobacteria* could provide defense against pathogens [100], and symbiotic *Cyanobacteria* might fix and provide nitrogen to the limpet [101].

## 3. Materials and Methods

### 3.1. P. pellucida Collection and Maintenance

Over 100 *P. pellucida* limpets were collected from the rocky seashore at Aberystwyth (52.4140°N, 4.0810°W), Ceredigion, Wales in late November 2012, when individuals were approximately 7 mm in length. Within one hour following collection, limpets were transferred to a glass tank filled with water of 3.2%–3.4% salinity. The tank was equipped with 2 sets of lights (AquaRay AquaBeam 600Ultra, Tropical Marine Centre, Bristol, UK) coupled to the timer (AquaRay Controller, Tropical Marine Centre) synchronised with the naturally occurring Mid-Wales (UK) light/dark cycle. The tank was connected to the Salt Water Filtration System (TMC System5000, Tropical Marine Center) supplying oxygen and providing re-circulated, temperature controlled, mechanically and biologically filtered, UV-sterilised seawater. A single water pump (SEIO Super Flow M250 Pump, TAAM, Camarillo, CA, USA) placed in the tank provided additional water movement. Each of the collected *P. pellucida* limpets was attached to the surface of *L. digitata*, submerged in the tank and left for one month to graze and grow. Maintenance of the limpets on *L. digitata* in the tank prior to dissection was to reduce transient bacteria, to ensure the limpets were large enough for accurate dissection and to make sure that they are actively grazing. Additionally, being maintained for one month prior to dissection ensured that if any bacteria essential for macroalgae breakdown were being lost from the limpets, then *P. pellucida* would not survive under these conditions.

### 3.2. Indirect Extraction of Metagenomic DNA

All the following extraction steps of indirect extraction of prokaryotic DNA from the *P. pellucida* digestive tract were conducted on ice unless otherwise stated. Sixty limpets were placed on sterile petri dishes at room temperature and anesthetized by flooding the plates with sterile, isotonic (7.2%) magnesium chloride solution [102]. After 15 min limpets were transferred to new, sterile petri dishes and sprayed with 70% ethanol to remove surface-contaminating microbes. Limpets were rinsed with distilled water and the intestines were then aseptically dissected. The dissected material was collected in 2 mL microcentrifuge tubes floating in liquid nitrogen, 1 mL of ice cold 50 mM potassium phosphate buffer at pH 7.5 was added, vortexed and homogenized for 2 s using homogenizer (T10 basic ULTRA-TURRAX, IKA-Werke GmbH & Co. KG, Staufen, Germany). Each sample was then centrifuged at 60× *g* for 30 s. One milliliter of supernatant from each sample was decanted into a new 2 mL microcentrifuge tube, passed through 90, 50 and subsequently 10 µm mesh cloths (Cadisch MDA Ltd., London, UK) and collected into new 2 mL microcentrifuge tubes. The fraction obtained was filtered again using a sterile 0.8 µm syringe filter (Gilson Scientific Ltd., Luton, UK) and collected into a 2 mL microcentrifuge tube. The final filtrate was centrifuged at 9600× *g* for 5 min to concentrate the microbial cells as a pellet. The supernatant was discarded and the pellet resuspended with 978 µL of 50 mM potassium phosphate buffer at pH 7.5. The DNA of this pellet was extracted using a FastDNA^®^ Spin Kit for Soil (Qbiogene, Cambridge, UK), according to the manufacturer’s protocol.

### 3.3. Metagenomic DNA Sequencing, Assembling and Taxonomic Profiling of the Corresponding Microbial Community

Indirectly extracted DNA was used to create an Illumina paired-end library with an average insert size of 360 base pairs (bp) according to the manufacturer’s instructions (TruSeq DNA LT Paired-End Sample Prep Kit, Rev. E, Illumina, Ltd., Essex, UK). The library was sequenced at 2 × 101 base pairs (bp) using the Illumina HiSeq2500 platform at the IBERS Aberystwyth Translational Genomics Facility (Aberystwyth, UK), according to standard procedures. The final sequenced library consisted of 398 million reads. The reads were cleaned using fastq-mcf from ea-utils [103], which removed adaptor sequences, low quality reads (a minimum quality score of 20 was required), and short reads <31 bp. The reads were then trimmed to remove the first 15 bp. This resulted in 391 million reads mostly in the size range of 78–84 bp, giving a total of 16.458 billion bp. These relatively short reads were additionally contaminated with host-derived sequences and so were not sufficient for reliable functional analysis. However they could be successfully used in taxonomic analysis of the microbiome. Taxonomic profiling of metagenomic DNA was performed using the MetaPhlAn (Metagenomic Phylogenetic Analysis) tool, which uses more than 115,000 prokaryotic clade-specific marker genes collected from over 1200 species [25]. It has been widely and successfully used in human microbiome studies where it has been proven to be capable of identifying taxonomies at the species level by aligning metagenomics reads to the database of marker genes in order to estimate the relative abundance of each microbial group [26]. Here we used MetaPhlAn with its default options *i.e.*, by using the bowtie short read aligner to map the reads against the database of marker genes. The advantage of using MetaPhlAn over *de novo* assembly approaches is that MetaPhlAn uses all reads available in the sample and can therefore identify species present at low levels, whereas *de novo* assembly approaches often struggle to generate contigs from these less abundant species with the result that they are often missed by downstream analyses [25]. In addition, MetaPhlAn has previously been positively evaluated on noisy shotgun reads derived from environmental samples with limited coverage of reference genomes. These features are relevant to this study because of contamination from the host (limpet). This species has not been sequenced and there are no suitable reference genomes that can be used to identify the limpet sequences. Hence it is not possible to remove the limpet sequences and as a result *de novo* assemblies are highly fragmented and difficult to analyze.

## 4. Conclusions

This study characterizes the metaphylogenome associated with the digestive tract of the seaweed grazing marine limpet *P. pellucida*. By modifying existing DNA extraction protocols, we indirectly extracted enough improved quality microbial DNA to create shotgun libraries, which were analyzed using prokaryotic, clade-specific marker genes. Metagenomic analysis of the microbiome harbored by the limpet indicated an abundance of industrially interesting bacterial groups (*Proteobacteria*, *Firmicutes*, and *Bacteroidetes*), previously identified in other seaweed- or sea grass-grazing animals. The phylogenetic profiles of these three phyla that were characterized through surveying the literature and their potential activities towards the host as well as applications to industry were assessed. Preliminary analysis of the microbial assemblage associated with *P. pellucida* demonstrated the great potential that these three phyla could offer in diverse biorefinery processes as well as the pharmaceutical or bioremediation industries. Future studies including functional metagenomics, metatranscriptomic and comparative genomics are expected to give further insight into the novelty of active bioproducts synthesized by members of this unique microbiome. These results could also benefit microbial ecology by extending the understanding of the relationship between microbes and their marine animal hosts.
